# In Situ Subcellular Detachment of Cells Using a Cell‐Friendly Photoresist and Spatially Modulated Light

**DOI:** 10.1002/advs.201900566

**Published:** 2019-05-17

**Authors:** Jeehun Park, Taeyup Kim, Jong Chul Choi, Junsang Doh

**Affiliations:** ^1^ School of Interdisciplinary Bioscience and Bioengineering (I‐Bio) Pohang University of Science and Technology 77, Cheongam‐ro Pohang Gyeongbuk 37673 South Korea; ^2^ Department of Mechanical Engineering Pohang University of Science and Technology 77, Cheongam‐ro Pohang Gyeongbuk 37673 South Korea; ^3^ Department of Materials Science and Engineering Seoul National University 1 Gwanak‐ro Gwanak‐gu Seoul 08826 South Korea

**Keywords:** cell detachment, cell migration, light‐responsive polymers, spatially modulated light

## Abstract

Dynamic adhesion and detachment of subcellular regions occur during cell migration, thus a technique allowing precise control of subcellular detachment of cells will be useful for cell migration study. Previous methods for cell detachment were developed either for harvesting cells or cell sheets attached on surfaces with low resolution patterning capability, or for detaching subcellular regions located on predefined electrodes. In this paper, a method that allows in situ subcellular detachment of cells with ≈1.5 µm critical feature size while observing cells under a fluorescence microscope is introduced using a cell‐friendly photoresist and spatially modulated light. Using this method, a single cell, regions in cell sheets, and a single focal adhesion complex within a cell are successfully detached. Furthermore, different subcellular regions of migrating cells are detached and changes in cell polarity and migration direction are quantitatively analyzed. This method will be useful for many applications in cell detachment, in particular when subcellular resolution is required.

## 1 Introduction

Regulated adhesion and detachment of cells is important for various processes in life, including development, homeostasis, wound healing, and immune responses.^1^ Engineered surfaces controlling cell adhesion have been developed for implantable devices, tissue engineering, and fundamental study of cell biology.^2^ A number of cell adhesive (e.g., RGD peptide) and cell repellent (e.g., poly(ethylene glycol (PEG)) moieties were identified and used to promote or prevent cell adhesion on surfaces.[qv: 2b,3] Furthermore, by controlling spatial distribution of cell adhesive and repellent moieties using microfabrication, morphologies, and spacing between neighboring cells were modulated.[qv: 2b,3,4] While these methods enabled precisely controlled cell–extracellular matrix (ECM)/cell–cell interactions to fine tune cell fates, differentiation, and activation,^5^ methods based on cell immobilization have limitations in recapitulating dynamic nature of life involving active migration and detachment of cells, which occurs under various physiological/pathological circumstances.^6^

To achieve dynamic modulation of cells on engineered surfaces, various stimuli‐responsive materials were used. Temperature‐responsive materials, including poly(*N*‐isopropylacrylamide) (PNIPAAm), have been widely used to detach cells by switching temperatures to harvest intact cell sheets and aggregates for further applications.^7^ However, spatially regulated cell detachment was challenging with temperature‐responsive materials. Electrical stimulation can be an alternative method for spatial modulation of cell adhesion and detachment,^8^ but this method requires predefined regions patterned with conducting materials, thus may not be as flexible as light stimulation. Near infrared (NIR) absorbing materials were used to selectively detach cells near light‐illuminated regions by converting light into heat.^9^ While NIR is a biocompatible light source with low toxicity, critical feature size/resolution achievable by this method is tens of micrometers, comparable to mammalian cell sizes, thus cannot be used for subcellular level control of cells. Ultraviolet (UV) light is attractive for high‐resolution light stimulation and direct photochemical conversion of chemical moieties, but can be toxic for cells.^10^

**Figure 1:**
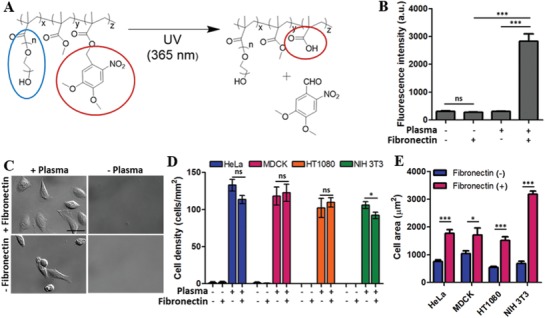
Characteristics of fibronectin‐coated PDMP. A) Photochemical reaction of PDMP. Blue circle: PEG side chain, red circle: organic soluble group (left side) to water soluble group (right side) conversion by the photochemical reaction. B) Effects of plasma treatment on the fibronectin adsorption on PDMP surfaces. The amount of fibronectin on the PDMP surfaces was measured by immunofluorescence microscopy. Fluorescence intensity is arbitrary unit (a.u.). C–E) Effects of plasma treatment and fibronectin coating of PDMP surfaces on cell adhesion. Cells seeded on each type of the surface were incubated for 3 h, washed to remove unbound cells, and DIC images were acquired. Representative DIC images of HeLa cells on different types of surfaces are shown in C (Scale bar: 50 µm). Using DIC images, D) cell density and E) cell area of four different cells (HT1080, MDCK, HeLa, and NIH 3T3) were measured on various types of surfaces. Data are shown as mean ± s.e.m. [two‐sided Student's *t*‐test] ns: not significant, *p < 0.05, *** *p*< 0.001.

To achieve high‐resolution control of cell dynamics with minimal cytotoxicity, our group developed a cell friendly photoresist poly(2,2‐dimethoxy nitrobenzyl methacrylate‐r‐methyl methacrylate‐r‐poly(ethylene glycol) methacrylate) (PDMP).^11^ PDMP undergoes photochemical reaction in response to 365 nm wavelength of light illumination (**Figure** **1**A) and becomes soluble in buffer solution with pH ≈ 7.4, including phosphate buffered saline (PBS) and standard cell culture media. Thin films of PDMP immersed in PBS or cell culture media are spontaneously dissolved by several seconds of brief light illumination through a 4′,6‐diamidino‐2‐phenylindole (DAPI) filter of a standard fluorescence microscope, a widely used light source for live cell imaging for cell nucleus, with minimal cytotoxicity.^12^ Original PDMP thin films are cell repellent due to the presence of PEG side chains,^11^, ^13^ thus by selectively removing PDMP thin films in certain regions by light illumination through a photomask, we can precisely control various dynamic cellular processes such as cell adhesion, spreading, and migration, and perform quantitative analysis.^12^, ^13^, ^14^ However, the previous methods primarily focused on triggering cell spreading and migration by removing PDMP thin films adjacent to cells to form new adhesion due to the cell repellent nature of PDMP. Methods that allow precise detachment of subcellular regions would be useful for the study of cell polarity and migration where subcellular adhesion plays an important role.^15^

In this study, we developed a new method that allows in situ detachment of cells with subcellular resolution using the cell friendly photoresist PDMP. First, we converted cell‐repellent properties of PDMP thin film surfaces to cell‐adherent by plasma treatment. Then, spatially modulated light (SML) generated by a digital micromirror device (DMD), which allowed fabrication of ≈1.5 µm features, was illuminated on cells adhering on surface‐modified PDMP to selectively detach single cells, multicellular clusters, and single focal adhesions. Using this new method, effects of different subcellular region detachment on cell polarity/migration were investigated.

## 2 Results and Discussion

### 2.1 PDMP Surface Modification

To create surfaces that initially promote cell adhesion, but can trigger partial detachment of cells by light illumination, the surfaces of PDMP, a cell friendly photoresist polymer previously developed for dynamic cell micropatterning (Figure 1A),^11^, ^12^, ^13^, ^16^ was modified. Original PDMP thin films have bioinert surfaces with minimal protein and cell attachment due to PEG side chains (a blue circle in Figure 1A).^11^ Therefore, additional surface treatments were required to coat adhesion molecules such as fibronectin on PDMP surfaces. To activate PDMP surfaces, they were treated with air plasma for 1 min prior to fibronectin coating. Physicochemical properties of untreated and plasma‐treated PDMP surfaces were extensively characterized by cross‐sectional scanning electron microscopy (SEM), water contact angle (WCA) measurement, atomic force microscopy (AFM), and X‐ray photoelectron spectroscopy (XPS), and shown in Figure S1 in the Supporting Information. Plasma treatment slightly etched PDMP thin films (cross‐sectional SEM) with almost no changes in surface roughness (AFM), and slightly increased hydrophilicity (WCA) with oxygen incorporation (XPS). Importantly, detailed analysis of C1s XPS peaks revealed substantial reduction of the C–O peak (≈286 eV) that are mostly generated from the (CH_2_CH_2_O)*_n_* occurred for plasma‐treated PDMP surfaces, indicating PEG side chains critical for protein resistance were damaged by plasma treatment. Fibronectin coating on the plasma‐treated PDMP surfaces was assessed by immunofluorescence microscopy (Figure 1B). In the absence of plasma treatment, undetectable amounts of fibronectin binding occurred (plasma‐/fibronectin + sample in Figure 1B), whereas at least tenfold increased fluorescence intensity was detected when the PDMP surfaces was treated with plasma prior to fibronectin coating (plasma + /fibronectin + sample in Figure 1B).

Next, we examined cell adhesion on the modified PDMP surfaces (Figure 1C). Four different types of cells, including HT1080 (human fibrosarcoma cell), MDCK (Madin–Darby canine kidney epithelial cell), HeLa (human cervical cancer cell), and NIH 3T3 (murine fibroblast), were used. Cells in cell culture media supplemented with 10% fetal bovine serum (FBS) were seeded on various surfaces for 3 h and gently washed to remove nonadhering cells. Then, differential interference contrast (DIC) images were acquired in randomly selected positions (Figure 1C), and average cell density was calculated (Figure 1D). In the absence of plasma treatment, no cell adhesion was observed for all cell types. In contrast, plasma treatment was sufficient to induce substantial cell adhesion in terms of cell density, presumably by promoting adhesion molecule binding in FBS on the plasma‐treated PDMP surfaces.^17^ However, cells on fibronectin‐coated PDMP surfaces exhibited more spread morphologies (Figure 1C) with significantly larger areas (Figure 1E) compared with cells on uncoated surfaces (or only plasma‐treated surfaces), meaning fibronectin coating on PDMP surfaces further enhanced cell adhesion.

### 2.2 In Situ Detachment of Cells on Fibronectin‐Coated PDMP Surfaces Using Spatially Modulated Light

**Figure 2:**
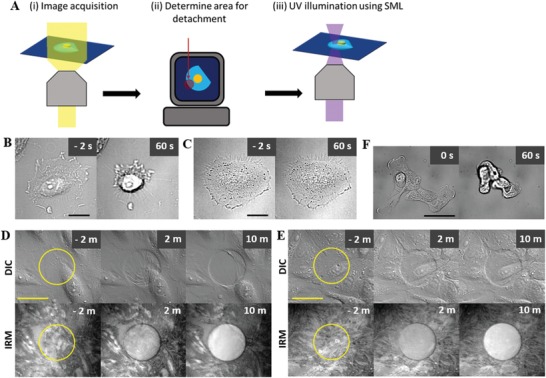
In situ detachment of cells using spatially modulated light (SML). A) Schematic procedure for in situ cell detachment. B,C) Representative DIC images of HeLa cells on a fibronectin‐coated B) PDMP or C) PMMA surfaces before (left) and after (right) SML illumination. Scale bar: 20 µm. Time at SML illumination is set to “0.” D,E) Representative time‐lapse DIC and IRM images of a D) HeLa and E) MDCK cell monolayers before and after SML illumination. SML illuminated regions were marked with yellow circles on DIC and IRM images acquired prior to SML illumination. Scale bar: 50 µm. Time at SML illumination is set to “0.” F) Representative MDCK cell cluster detached by SML illumination. Scale bar: 50 µm. Time at SML illumination is set to “0.”

In situ detachment of cells adhering on fibronectin‐modified PDMP thin films was achieved by following procedure schematically shown in **Figure** **2**A: 1) a digital image of cells was acquired (Figure 2Ai), 2) a region for detachment was defined in the digital image (Figure 2Aii), and 3) PDMP thin films underneath the cell in the predefined regions were dissolved by illuminating spatially modulated light (SML, Figure 2Aiii). In order to implement this procedure, we integrated a DMD to a fluorescence microscope (Figure S2, Supporting Information).^18^ Each micromirror in the DMD can be titled to two different angles, thus we can generate a beam with a desired shape by adjusting the tilting angle of each mirror. Using this instrumental setup, we next tested whether we can perform micrometer‐scale micropatterning on fibronectin‐coated PDMP thin films by illuminating SML with an array of circles with various diameters (1.5–10 µm). Dissolution of PDMP thin films and generation of fibronectin micropatterns were confirmed by DIC and fibronectin immunofluorescence microscopy for all diameters of SML (Figure S3, Supporting Information). Therefore, we could generate micropatterns with critical feature size of 1.5 µm, which corresponds to subcellular length scale, on fibronectin‐coated PDMP surfaces.

With this micropatterning capability, we next tested whether we can detach cells attached on fibronectin‐coated PDMP surfaces by dissolving the PDMP thin films underneath the cells by SML illumination. First, SML completely covering single HeLa cells attached on the fibronectin‐coated PDMP surfaces was illuminated for 3 s, and behaviors of the HeLa cells were observed by time‐lapse microscopy (Figure 2B; Movie S1, Supporting Information). HeLa cells, initially spread on the surfaces with flat morphologies, rounded up with peripheral dark rings in DIC images, which occurs when cells were slightly out of focus,^19^ meaning cells were detached from the surfaces by SML illumination. To rule out the possibility that cell detachment occurred by brief light illumination, identical experiments were performed using fibronectin‐coated poly(methyl methacrylate) (PMMA) surfaces, which has identical backbone structure to PDMP but lacks photoresponsive moiety. As expected, no detectable detachment was observed for cells on PMMA surfaces, even with 10 s of SML illumination (Figure 2C; Movie S2, Supporting Information), suggesting cell detachment on PDMP surfaces was due to the dissolution of light illuminated‐PDMP thin films, not due to direct light‐mediated adverse effects on cells.

Next, we observed detachment behaviors of cells forming confluent monolayers. To clearly observe dissolution of PDMP underneath cell monolayers, interference reflection microscopy (IRM) images, which generates dark spots for regions with cell–substrate contact <100 nm,^20^ were acquired in conjunction with DIC images. Behaviors of detached cells varied for different types of cells presumably due to differences in cell–cell adhesion strengths.^14^ When we detached cells in the middle of monolayers of HeLa cells, which tends to form weak cell–cell junctions, by illuminating circular SML with 50 µm diameter, the majority of junctions between detached cells were broken, and the detached cells were drawn toward undetached cells (Figure 2D; Movie S3, Supporting Information). In sharp contrast, when we performed identical experiments with MDCK cells, which forms tight cell–cell junctions, junctions in the detached cells remained intact, and the detached cells remained on the SML‐illuminated region with substantial relocation (Figure 2E; Movie S4, Supporting Information). IRM images confirmed that PDMP thin film on the SML‐illuminated region was dissolved, but MDCK cells on the PDMP‐dissolved region remain suspended on the surfaces as shown in DIC images, presumably by tight cell–cell junctions. Using this property of MDCK cells, we could harvest intact small‐sized multicellular clusters of MDCK cells by illuminating SML covering entire multicellular clusters (Figure 2F; Movie S5, Supporting Information). The capability of detaching single cells and multicellular clusters can be useful for harvesting specific cells in cell mixtures if they adhere onto fibronectin‐coated PDMP surfaces.

### 2.3 Detachment of a Focal Adhesion Complex in a Cell

**Figure 3:**
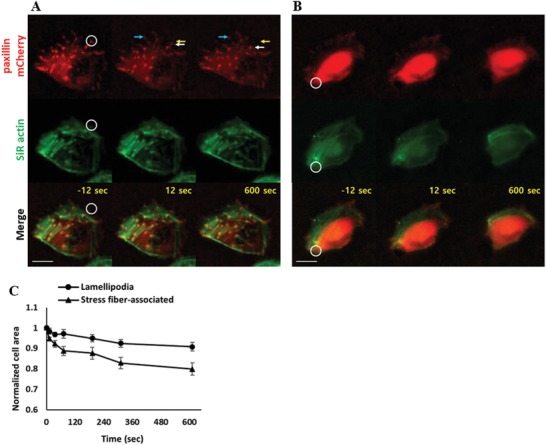
Detachment of focal adhesion complex (FAC). A,B) Representative time‐lapse images of FAC‐detached cells. FAC located A) in lamellipodia and B) associated with stress fibers located in the cell side were detached. FAC was labeled with paxillin–mCherry and F‐actin was labeled with an SiR actin probe. White circles are SML illuminated regions. White arrows: detached FACs. Yellow arrows: initial position of the detached FAC. Blue arrows: undetached FACs. Scale bar: 20 µm. Time at SML illumination is set to “0.” C) Effects of FAC detachment on cell area. FACs located in lamellipodia or associated with stress fiber were detached, and normalized cell areas, defined by cell area at time *t* divided by initial cell area, of cells after cell detachment were measured and plotted. Data are shown as mean ± s.e.m. of five cells in each condition.

As the critical feature size for our patterning technique is close to the size of a focal adhesion complex (FAC),^21^ we next attempted to detach a single FAC. HeLa cells transfected with paxillin–mCherry were used to visualize FACs,^22^ and filamentous actin (F‐actin) was stained using an SiR actin probe.^23^ FACs are dynamic supramolecular assemblies of macromolecules connecting integrins bound to extracellular substrates and F‐actin. Therefore, we thought by selectively dissolving regions underneath certain FACs, we could detach cell adhesions mediated by the specific FACs. We illuminated light on a circular spot with 3 µm diameter (white circles in **Figure** **3**A,B) that covered a FAC using SML to selectively dissolve fibronectin‐coated PDMP films underneath the FAC, and monitored behaviors of cells and FACs by time‐lapse microscopy. Interestingly, FACs detached by SML translocated inward to the cell body rather than disassembled for the majority of the cases (13 out of 15, Figure 3A; Movie S6, Supporting Information). Force sensing and transmission occurs through FACs,^24^ thus detachment of integrin associated with certain FACs could lead to the movement of FACs toward cell bodies to release tensions applied to the FACs via F‐actin. When FACs were connected with stress fibers, which generated strong F‐actin fluorescence signals due to the formation of bundles of F‐actin and myosin II, detachment of the FACs from the substrates leads to substantial shrinkage of the cells (Figure 3B; Movie S7, Supporting Information) due to the release of traction forces mediated by myosin II in stress fibers.[qv: 24a,b,25] Indeed, FACs in lamellipodia, which were not associated with stress fibers, could be detached with minimally influencing cell adhesion area (≈5%), whereas FACs located at cell sides slightly backward of lamellipodia, which were associated with stress fibers, were detached with substantial cell shrinkage (≈20%, Figure 3C).

### 2.4 Influence of Subcellular Detachment on Cell Polarity and Migration

In the previous section, FACs at the cell peripheries were visualized by paxillin–mCherry and selectively detached using SML illumination. Visualization of entire FACs in the cells was technically challenging with a wide‐field fluorescence microscope used in our experimental setting due to limited resolution and overwhelming fluorescence signals of paxillin–mCherry in cytoplasm that were not associated with FACs as shown in Figure 3B.^26^ Therefore, instead of detaching a specific FAC, we detached adhesions in a specific subcellular region in a polarized and migrating cell, and observed how cell polarity and migration were affected by the local detachment. Indeed, different types of FACs exist in a polarized cell depending on the subcellular regions: nascent FACs formed in the lamellipodia with leading edge protrusion gradually grow and mature as they move backward of the cells by cell migration.[qv: 1c,27] Therefore, partial detachment of a certain subcellular region is likely to alter cell polarity and migration by influencing force balance within a cell.

**Figure 4:**
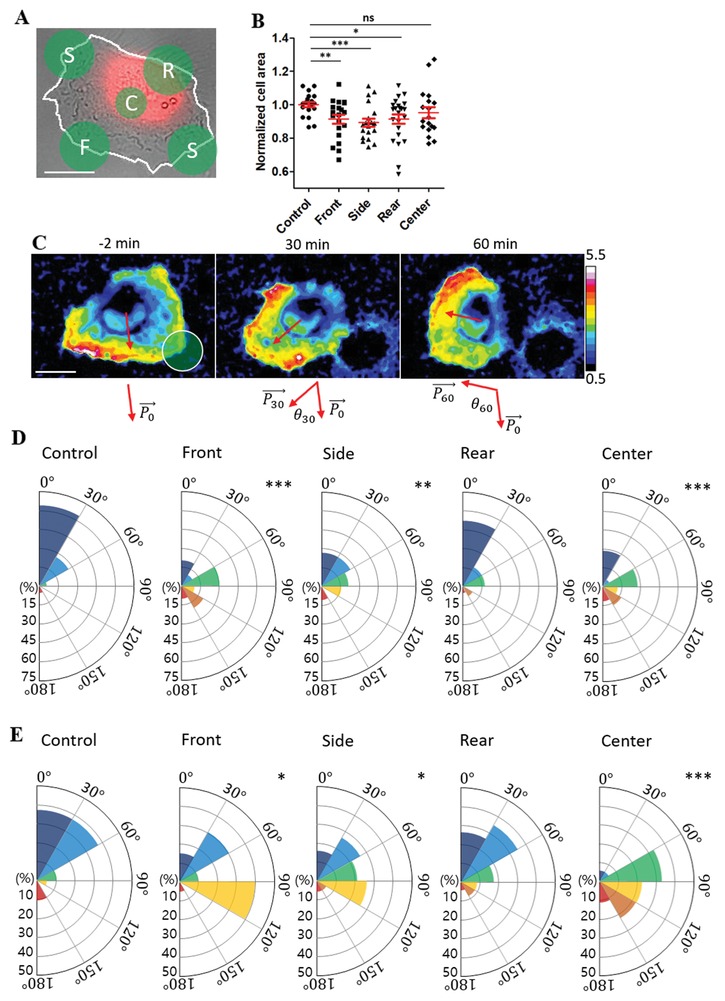
Effects of subcellular region detachment on cell polarity and migration. A) Representative DIC/CellTrace Far Red (CTFR) overlay image marked with different regions for detachment. White line: cell boundary. F: front; S: side; R: rear; C: center. Scale bar: 20 µm. B) Normalized cell area of subcellular detached cells. Normalized cell area was measured by the ratio of cell area 10 min after detachment and cell area 2 min before detachment. Two‐sided Student's *t*‐test was used. C) Normalized time‐lapse PH‐Akt‐YFP images with PIP_3_ orientation (red arrows) for a front‐detached cell. Time at SML illumination is set to “0.” Scale bar: 20 µm. D) Distribution of PIP_3_ orientation change for control (undetached) and front/side/rear/center detached cells at 60 m after detachment. E) Distribution of migration direction change for control (undetached) and front/side/rear/center detached cells at 60 m after detachment. 18–23 cells in each case were analyzed, and Kolmogorov–Smirnov test D,E) was used for statistical analysis. *p < 0.05, **p < 0.01, ***p < 0.001.

To identify and trace cell polarity, PH‐Akt‐YFP, which probe phosphatidylinositol (3,4,5)‐trisphosphates (PIP_3_) distribution on the cell membrane,^28^ was transfected into HeLa cells. Phosphoinositide 3‐kinases (PI3K) activity at the leading edge of migrating cells generates PIP_3_ at the plasma membrane to modulate cytoskeleton organization for membrane protrusion,[qv: 1a,29] thus PIP_3_ is a good marker for cell front. PH‐Akt‐YFP‐transfected HeLa cells were further labeled with CellTrace Far Red (CTFR), which labels cell cytoplasm. Based on DIC and CTFR images, front/rear of the migrating cells were identified: typically, migrating HeLa cells on fibronectin‐coated PDMP surfaces exhibited half‐moon shape morphology with arc at the cell front by thin membrane lamellipodia formation and thick cytoplasm located at the cell center/rear (**Figure** **4**A). Then, regions for front/side/rear/center detachment were determined as schematically shown in Figure 4A. For center detachment, a circular region of diameter 14 µm was used, whereas for front/side/rear detachment, a circular region of diameter 20 µm that overlap with ≈50% of cells, thus detach effectively the same area as the center region, was used. Time‐lapse imaging of DIC/PH‐Akt‐YFP/CTFR was performed before and after partial cell detachment to monitor cell shape/polarity/migration. PIP_3_ orientation and migration direction were determined using the PH‐Akt‐YFP and CTFR images by following the procedure described in Figures S4 and S5 in the Supporting Information, respectively. Front/side/rear detachment significantly reduced cell areas, whereas center detached cells exhibited comparable cell area to control (undetached) cells (Figure 4B), indicating detachment of cell peripheries induce cell shrinkage regardless of regions due to release of adhesion‐mediated traction forces. Time‐lapse images of normalized PH‐Akt‐YFP images (obtained by the ratio of PH‐Akt‐YFP images and CTFR images) for a front‐detached cell along with PIP_3_ orientation at different time points (red arrows in Figure 4C; Movie S8, Supporting Information) clearly showed drastic changes in PIP_3_ orientation and migration direction by partial cell detachment. PIP_3_ orientation change at *t* min after partial cell detachment was measured by angle differences between initial PIP_3_ orientation ($\vec{P_{0}}$) and PIP_3_ orientation at time *t* ($\vec{P_{t}}$), defined as θ_*t*_ in Figure 4C, and the distribution of θ_30_ and θ_60_ for control (undetached) and front/rear/side/center detached cells were plotted in Figure S6 in the Supporting Information and Figure 4D, respectively. In addition, migration direction change at time *t*, ϕ_*t*_, was measured by measuring angle between $\vec{P_{0}}$ and migration direction at *t*, and distributions of ϕ_30_ and ϕ_60_ for different cell detachment regions were plotted in Figure S7 in the Supporting Information and Figure 4E, respectively. In the absence of partial detachment (control), the majority of cells maintained initial polarity and minimally changed migration direction over 1 h. Rear detachment minimally affected PIP_3_ orientation and migration direction, whereas front/side/center detachment significantly altered PIP_3_ orientation and migration direction. Changes in PIP_3_ orientation and migration direction occurred gradually for the majority of cells, similar to the case of the front‐detached cells shown in Movie S8 in the Supporting Information, rather than abruptly. Migration direction change can be induced by changes in protrusion direction as well as shrinkage of cells mediated by detachment, whereas PIP_3_ orientation change reflect altered polarity within cells due to partial detachment. Therefore, migration direction change and PIP_3_ orientation change correlated, but did not completely agree with each other. Perturbation in force balances in lamellipodia/lamella regions located in front/side of migrating HeLa cells may be a major cause for PIP_3_ orientation and migration direction changes for front/side detached cells. In addition, prevention of new adhesion formation on detached regions, as shown in IRM images in Figure 2D,E, may bias new adhesion sites to alter cell polarity and migration, as center detachment caused substantial changes in PIP_3_ orientation and migration direction without any significant cell shrinkage. While detailed mechanisms need to be further investigated, potentially by performing high‐resolution fluorescence microscopy of detached components and polarity‐regulating molecules in conjunction with subcellular detachment, our method enabled us to precisely detach subcellular regions and quantitatively analyze cell behaviors after detachment.

Previously, subcellular regions of migrating fibroblasts were detached by locating a pipet tip releasing a cell adhesion peptide GRGDTP either near front or rear of cells, and changes in cell area, traction force, and migration direction were monitored.[qv: 6b] Overall, the results of the previous study agreed well with ours: rear detachment caused substantial changes in cell area, and minimal changes in traction force and migration direction, whereas prolonged exposure of GRGDTP peptide near front caused changes in cell polarity and migration direction. However, precise control of cell detachment using a pipet tip is technically challenging, and spatial resolution of cell detachment is also limited. In contrast, our method allows us to detach precisely controlled subcellular regions, including regions within central areas of cells.

Cell detachment techniques based on thermos‐sensitive polymer layers,^7^ and NIR‐sensitive thin films^9^ were developed, but their applications were mostly detachment of cell layers or small cell clusters due to limited resolution. Electrochemical release of adhesion molecules attached on electrodes can be used for high‐resolution subcellular region detachment,^8^ but in this case, only regions predefined by electrodes can be detached. Compared with the previous methods for cell detachment, our method is superior in that we can achieve high‐resolution in situ subcellular region detachment by using SML and light‐responsive cell‐friendly photoresist PDMP.

## 3 Conclusion

We developed a new cell detachment method that allowed in situ detachment subcellular regions of cells using a cell friendly photoresist PDMP and SML. To achieve this goal, we first modified PDMP surfaces by plasma treatment to allow cell adhesion on PDMP surfaces. Then, we integrated a DMD to a wide‐field fluorescence microscope to generate SML that allowed us to generate micropatterns with critical size ≈ 1.5 µm, comparable to the size of a single FAC. Using this new method, we demonstrated detachment of a single cell, cells in monolayers, and a single FAC. In addition, we investigated how different subcellular region detachment influenced cell polarity and migration. Our method will be useful for wide applications for cell detachment, in particular for the cases when high‐resolution in situ partial cell detachment is required.

## 4 Experimental Section

*Fluorescence Microscopes*: A modified Zeiss Axio Observer Z1 epifluorescence microscope with 40X (Plan‐Neofluar, NA = 1.30) and 20X (Plan‐Neofluar, NA = 0.5) objective lens and a Roper Scientific CoolSnap HQ charge‐coupled device (CCD) camera was used for fibronectin intensity measurement and cell adhesion assay. An XBO 75 W/2 xenon lamp (75 W, Osram) and GFP filter (EX BP 470/40, BS 495, EM BP 525/50) was used for fluorescence imaging for fibronectin intensity measurement. A modified Olympus IX 81 epifluorescence microscope with a 40X (UPlanFLN, NA = 1.30) objective lens and a Roper Scientific Cascade camera was used for cell detachment and imaging experiments. A X‐Cite series 120 PC lamp (120 W, Excelitas) and DAPI filter (EX 365) were used for SLM. A LAMBDA LS xenon lamp (175W, Sutter instrument) and Texas Red (EX BP 559/34, BS 580, EM BP 630/69), Cy5 (EX BP 620/60, BS 660, EM BP 770/75) filter sets were used for fluorescence imaging after cell detachment. Both microscopes were automatically controlled by Metamorph, and stages were equipped with Chamlide TC incubator system (Live Cell Instrument, Korea) to maintain a cell culture condition (37 °C, CO_2_ 5%).

*Fibronectin‐Coated PDMP Thin Film Preparation*: Random terpolymer PDMP was synthesized and characterized as described elsewhere.^11^ Clean coverslips were coated with gelatin (Sigma) by incubating in a 0.1% gelatin solution at room temperature for 30 min. Gelatin coated coverslips were spin coated with 3 wt% of PDMP in 1,4‐dioxane (Sigma) at 2000 rpm for 2 min, and baked at 100 °C for 24 h. PDMP thin films were treated with air plasma using CUTE (Femto Science) for 1 min and coated with fibronectin by incubating in fibronectin solution (50 µg mL^−1^ in PBS) at 37 °C for 30 min. Fibronectin coating was validated by immunofluorescence microscopy using anti‐fibronectin rabbit antibody (EMD millipore, polyclonal) as a primary antibody and anti‐rabbit goat antibody tagged with Alexa fluor 488 (Abcam, polycolnal) as a secondary antibody.

*Cell Culture and Cell Adhesion Assay*: HeLa, MDCK, HT1080, and NIH‐3T3 cells were cultured in Dulbecco's Modified Eagle's medium (DMEM) (Gibco) supplemented with 10% FBS (Gibco), 1% penicillin–streptomycin (Gibco). Cell suspension (1 mL, 5 × 10^4^ cells mL^−1^) was applied on the PDMP surfaces and incubated for 2 h. Then, unattached cells were washed with PBS, and the surfaces were mounted on a microscope. DIC images of 10 randomly selected positions were acquired with a 20X objective lens, and number of cells in each position was manually counted and converted to cell density.

*DNA Preparation and Transfection*: Paxillin–mCherry (addgene) and PH‐Akt‐YFP (gift from Prof. Sung Ho Ryu in POSTECH) plasmid DNA was prepared by maxi‐prep kit (Qiagen) and concentrated to 1 mg mL^−1^. Neon transfection system (Invitrogen) was used for DNA transfection. Briefly, DNA (8 µL) was added to HeLa cell suspension (10^6^ cells) in 150 µL of R buffer (Invitrogen). Then, the cell and DNA mixture was loaded in 100 µL electroporation tip (Invitrogen) and 1 electric pulse was treated with 1400 V for 20 ms. Transfected cells were seeded in cell culture dishes filled with DMEM cell growth medium containing 10% FBS and 1% P/S, and cultured for 1 day in an incubator maintaining 37 °C, 5% CO_2_.

*Instrumentation for Spatially Modulated Light Generation*: Spatially modulated light (SML) was generated by the optical system schematically shown in Figure S2 in the Supporting Information. A DMD (DLP Discovery 4100 Development Kit, Texas instrument) was located at a diaphragm of a fluorescence microscope (Figure S2, Supporting Information). Light from a light source (Metal Halide, 120 W) was adjusted to reach the DMD after total reflection by the total internal reflection (TIR) prism. SML generated by the DMD passed through the TIR prism and was reflected by a dichroic mirror to an objective lens. MATLAB and ALP basic GUI (ViALUX GmbH) were used to control DMD to determine the shape of SML.

*Cell Detachment Experiments*: Cells were seeded on fibronectin‐coated PDMP surfaces, incubated in a cell culture incubator (37 °C, 5% CO_2_) for at least 3 h, and mounted on a microscope stage equipped with an incubator (37 °C, 5% CO_2_). Digital images of the cells were acquired to select a region for detachment. The selected region was briefly illuminated with SML using a DMD, and time‐lapse imaging was initiated. F‐actin was labeled by adding 500 × 10^−9^ m of an SiR‐actin (Cytoskeleton, Inc.) in the media, and cytoplasm was labeled using CTFR (Invitrogen) by following the manufacturer's instruction.

*Image Analysis*: Acquired images were analyzed using Image J (NIH). Cell area and center of mass of the cells were measured by manually drawing cell boundaries in DIC images. PIP_3_ orientation and cell migration direction was measured using PH‐Akt‐YFP transfected cells labeled with CTFR as described in Figures S4 and S5 in the Supporting Information.

## Conflict of Interest

The authors declare no conflict of interest.

## Supporting information

Supplementary

Supplementary

Supplementary

Supplementary

Supplementary

Supplementary

Supplementary

Supplementary

Supplementary
