# Supplementary material for: In Situ Subcellular Detachment of Cells Using a Cell‐Friendly Photoresist and Spatially Modulated Light
Source: Adv Sci (Weinh). 2019 May 17;6(14):1900566. doi: 10.1002/advs.201900566 (PMC6661940; doi:10.1002/advs.201900566)
Supplement: Supplementary file 1 — Supplementary [file ADVS-6-1900566-s002.pdf]

Copyright WILEY-VCH Verlag GmbH & Co. KGaA, 69469 Weinheim, Germany, 2019.

ADVANCED SCIENCE
Open Access

# Supporting Information

for *Adv. Sci.,* DOI: 10.1002/advs.201900566

## In Situ Subcellular Detachment of Cells Using a Cell-Friendly Photoresist and Spatially Modulated Light

*Jeehun Park, Taeyup Kim, Jong Chul Choi, and Junsang Doh**

Copyright WILEY-VCH Verlag GmbH & Co. KGaA, 69469 Weinheim, Germany, 2019.

# Supporting Information (SI)

## In situ subcellular detachment of cells using a cell-friendly photoresist and spatially modulated light

*Jeehun Park, Taeyup Kim, Jong Chul Choi, and Junsang Doh**

### Supporting Figures

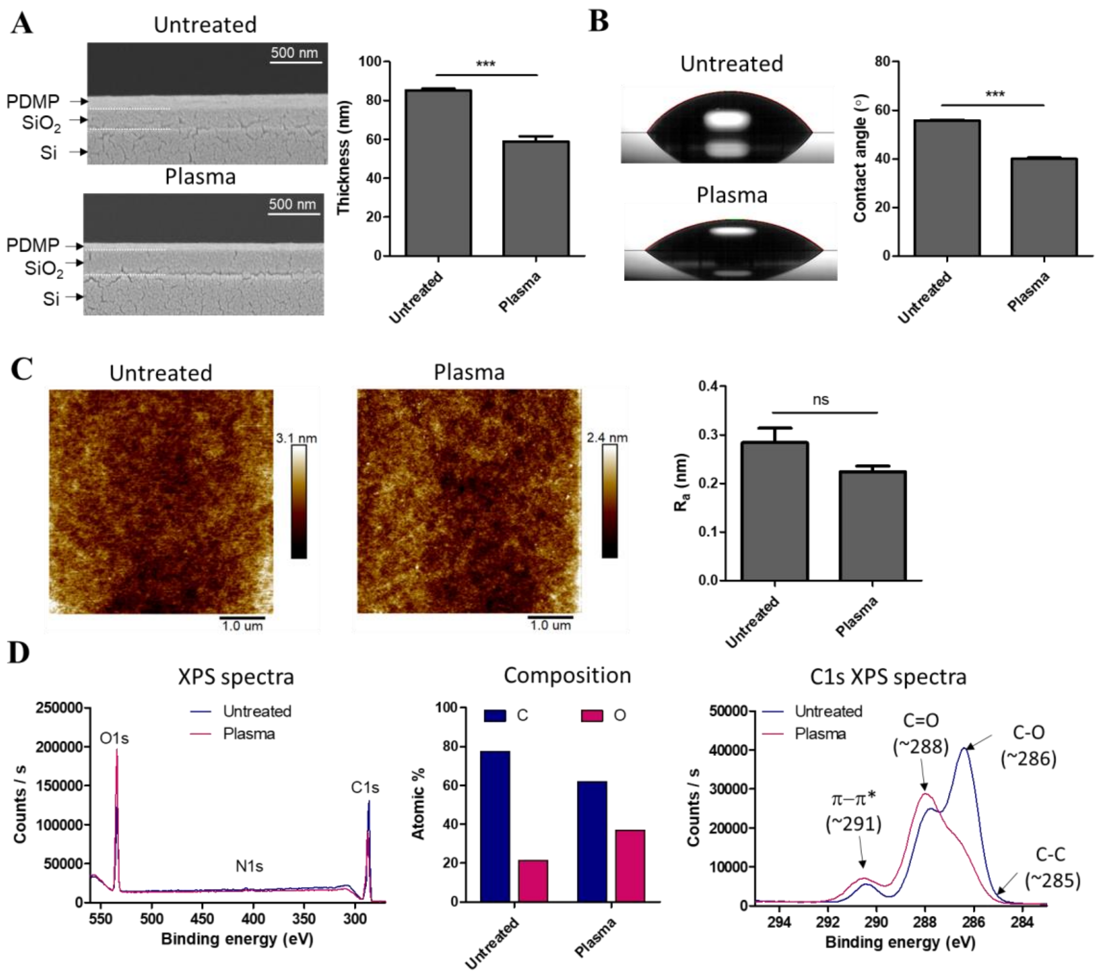


**Figure S1**. Characterization of untreated and plasma-treated PDMP thin films. **A.** Film thicknesses were measured by crosssectional SEM using Philips XL30S (FEI). **B.** Water contact angles were measured using Smart drop (Femtofab). **C.** Average roughness ($R_a$) of surfaces were measured by atomic force microscopy (AFM) using Dimension 3100+ nanoscope V (VEECO). **D.** Surface chemistry analysis was performed by X-ray photoelectron spectroscopy (XPS) using XSAM 800 cpi ESCA (KRATOS Analytical). Each peak of the O1s and C1s peaks in XPS spectra (left panel) was integrated to obtain composition of C and O (middle panel). C1s XPS spectra area was zoomed in and each peak was assigned with the corresponding chemical bond (right panel).

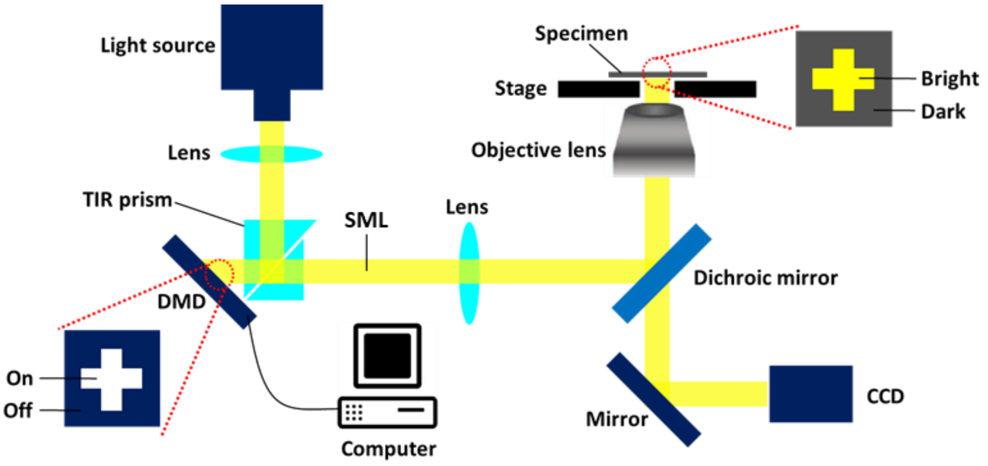


**Figure S2**. Schematic illustration of a spatial light modulator setup integrated in a fluorescence microscope. Digital micro-mirror device (DMD) was located at a diaphragm of a fluorescence microscope. Light from the light source is adjusted to reach the DMD after total reflection in the TIR prism. Spatially modulated light (SML) generated by the DMD will pass through the TIR prism, and be reflected by the dichroic mirror located below an objective lens to reach a specimen loaded on a microscope stage. Matlab and ALP basic GUI (ViALUX GmbH) are used to control DMD.

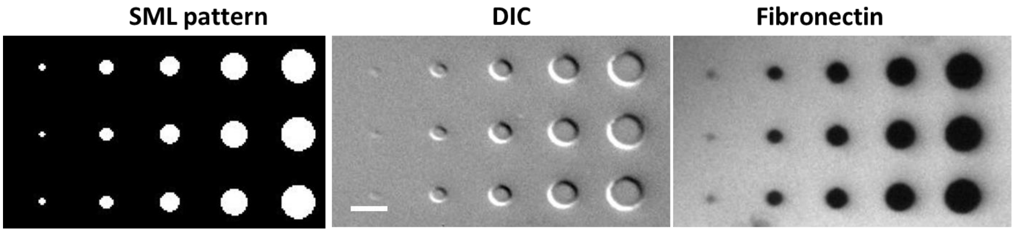


**Figure S3**. Micropatterning of fibronectin-coated PDMP surfaces to determine critical feature size. Fibronetcin-coated PDMP thin films immersed in phosphate buffered saline (PBS, pH: 7.4, 10 mM phosphate buffer, 2.7 mM potassium chloride, 137 mM sodium chloride) solution was loaded on a microscope stage, and spatially modulated light (SML) with an array of circles with various diameters (1.5 ~ 10 μm) was illuminated for 3 s. Dissolution of PDMP films exposed to the SML was evident by a DIC image. Removal of fibronectin coated on the PDMP thin films was further confirmed by immunofluorescence microscopy for fibronectin. Scale bar: 10 μm

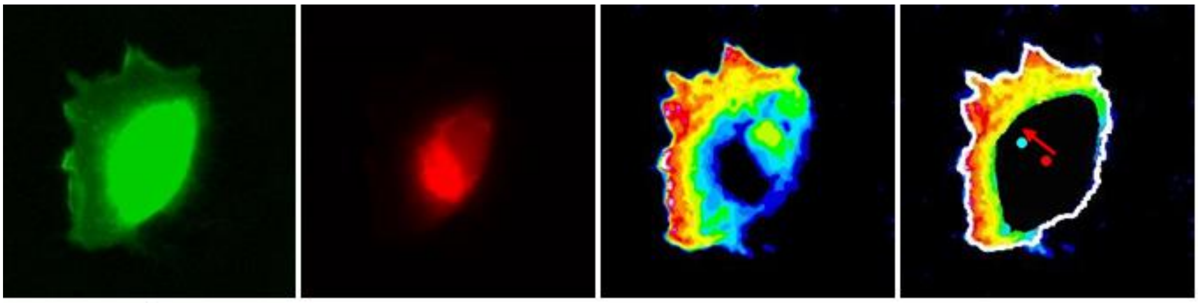


**Figure S4**. Determination of $PIP_3$ orientation using PH-Akt-YFP and Cell tracer Far-Red (CTFR) fluorescence images. PH-Akt-YFP transfected cells were labeled with CTFR and used for imaging. PH-Akt-YFP and CTFR fluorescence images of migrating cells were acquired and processed after background subtraction. 'Normalized PH-Akt-YFP' image was obtained by taking the ratio of PH-Akt-YFP image and CTFR image. Since we are interested in $PIP_3$ distribution near cell periphery, fluorescence signals in cell cytoplasm, defined by the region with top 85% CTRF fluorescence intensity, was removed to obtain 'normalized and cytoplasm subtracted PH-Akt-YFP' image. In normalized and cytoplasm subtracted PH-Akt-YFP image, center of cytoplasm is marked with a red dot and intensity-weighted centroid of normalized and cytoplasm subtracted PH-Akt-YFP is marked with a cyan dot, and a vector connecting the red dot and the cyan dot is defined as a vector for $PIP_3$ orientation (red arrow).

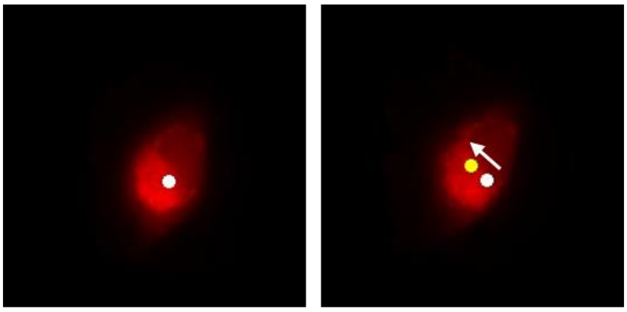


**Figure S5**. Determination of migration direction using Cell tracer Far-Red (CTFR) fluorescence image. Centroid of cytoplasm, defined by the region with top 85 % fluorescence intensity, at t = 0 is marked with white dots, and centroid of cytoplasm at time t is marked with a yellow dot. Migration direction at time t is defined by a vector connecting a white dot and a yellow dot (white arrow).

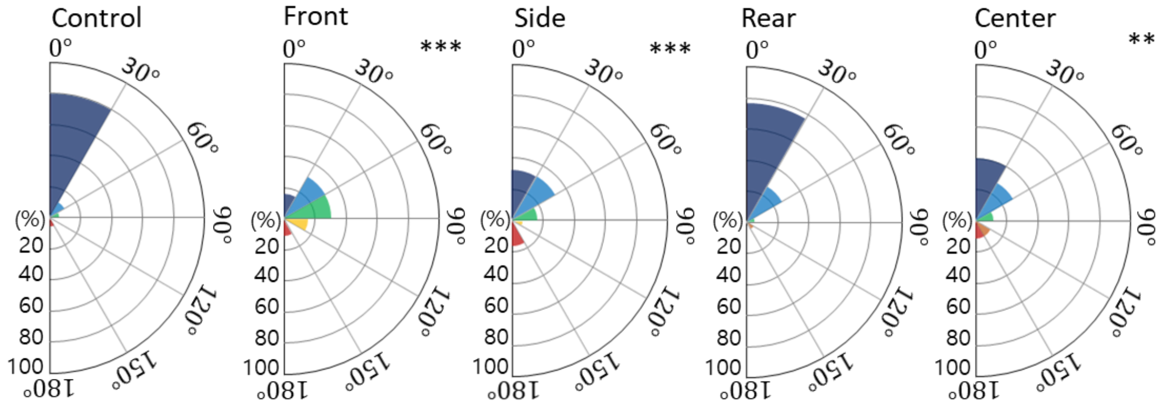


**Figure S6**. Distribution of $PIP_3$ orientation change for control (undetached) and front/side/rear/center detached cells at 30 m after detachment. Kolmogorov–Smirnov test was used for statistical analysis. ** $p < 0.01$, *** $p < 0.001$.

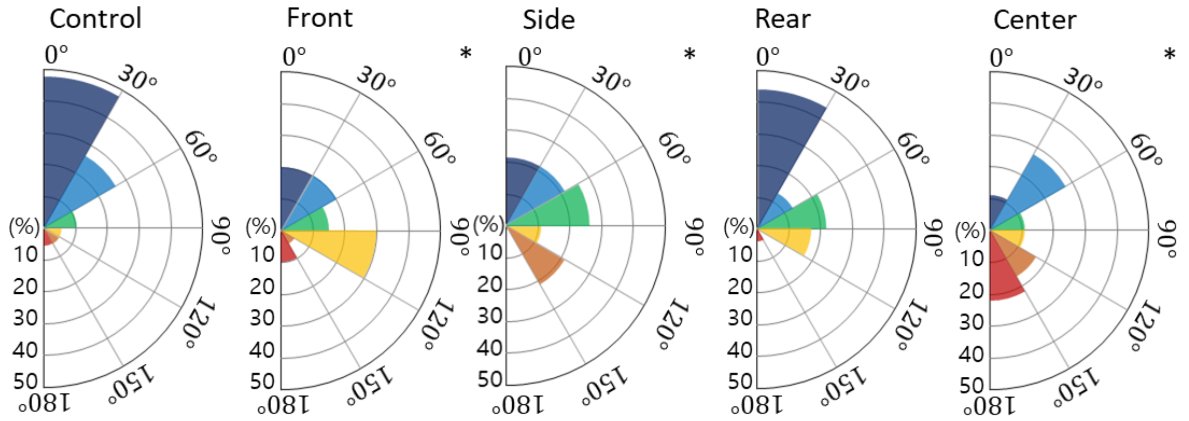


**Figure S7**. Distribution of migration direction change for control (undetached) and front/side/rear/center detached cells at 30 m after detachment. Kolmogorov–Smirnov test was used for statistical analysis. * $p < 0.05$.

**Supporting Moive Legends**

**Movie S1.** Representative DIC movie of a HeLa cell on fibronectin-coated PDMP detached by a spatially modulated light (SML). SML was illuminated to the blue area at time '0' for 3 s. Scale bar: 20 μm.

**Movie S2.** Representative DIC movie of a HeLa cell on fibronectin-coated PMMA. Spatially modulated light (SML) was illuminated to the blue area at time '0' for 10 s. Scale bar: 20 μm.

**Movie S3.** Representative DIC (left)/IRM (right) composite movie of HeLa cell monolayer detached by spatially modulated light (SML). SML was illuminated to the blue area at time '0' for 3 s. Scale bar: 50 μm.

**Movie S4.** Representative DIC (left)/IRM (right) composite movie of MDCK cell monolayer detached by spatially modulated light (SML). SML was illuminated to the blue area at time '0' for 3 s. Scale bar: 50 μm.

**Movie S5.** Representative DIC movie of a MDCK cell cluster detached by spatially modulated light (SML). SML was illuminated to the entire area at time '0' for 3 s. Scale bar: 50 μm.

**Movie S6.** Representative fluorescence movie of a HeLa cell detached with a focal adhesion complex (FAC) in lamellipodia. Spatially modulated light (SML) was illuminated to the area in a white circle at time '0' for 3 s. Red: paxillin-mCherry. Green: F-actin. Scale bar: 20 μm.

**Movie S7.** Representative fluorescence movie of a HeLa cell detached with a focal adhesion complex (FAC) in lamella associated with F-actin. Spatially modulated light (SML) was illuminated to the area in a white circle at time '0' for 3 s. Red: paxillin-mCherry. Green: F-actin. Scale bar: 20 μm.

**Movie S8.** Representative normalized PH-Akt-YFP fluorescence movie of a front-detached HeLa cell. Spatially modulated light (SML) was illuminated to marked area at time '0' for 3 s. $PIP_3$ orientation was marked with red arrows. Scale bar: 20 μm.
